# Editorial: Identification of New Molecular Mechanisms of Bone Disease, volume II

**DOI:** 10.3389/fcell.2023.1166647

**Published:** 2023-03-07

**Authors:** Amelie Coudert, Giulia Battafarano, Andrea Del Fattore

**Affiliations:** ^1^ UFR d’odontologie (Département des Sciences Biologiques), Université Paris Diderot BIOSCAR-INSERM U1132, Paris, France; ^2^ Bone Physiopathology Research Unit, Translational Pediatrics and Clinical Genetics Research Division, Bambino Gesù Children’s Hospital, IRCCS, Rome, Italy

**Keywords:** rare bone diseases, bone serum biomarkers, bone remodeling biomarkers, bone diseases markers, bone diseases targets

In the last years, the identification of new biomarkers to perform a correct and opportune diagnosis of bone diseases has been a great interest in the field. Although many bone biomarkers have been identified, so far only two serum makers have been recognized as relevant for diagnostic purposes: CTX (C-terminal telopeptide of type I collagen) as a marker of bone resorption and P1NP (N-terminal propeptide of type I procollagen) as sign of bone formation (Vasikaran et al., 2011). However, the measurement of these two molecules is not sufficient to perform early diagnosis and to set a therapeutic plan for some bone diseases.

Indeed, the lack of effective biomarkers makes difficult to achieve the prompt diagnosis and intervention for osteonecrosis of the femoral head (ONFH). In their paper here published, Liu et al. described the lncRNA (Long Non-coding RNA) growth arrest-specific transcript 5 (GAS5) as a potential biomarker for ONFH early diagnosis. The authors found that the expression of lncRNA GAS5 was significantly downregulated in patients with ONFH; this result was also confirmed in a rat model of ONFH. The relevance of lncRNA GAS5 in the bone remodeling activity was demonstrated by its increased expression during osteoblast differentiation and by its downregulation in osteoporosis patients and mice (Feng et al., 2019; Li et al., 2020). The key role of long non-coding RNA in the regulation of bone remodeling was also described in the paper published by Hu et al.; in their study, the authors identified an upregulation of Gm15222 and a positive correlation between its levels and BMP4 in diet induced obese (DIO) mice. Moreover, they demonstrated that Gm15222 promotes osteogenesis and inhibits the expression of adipogenesis-related genes in DIO by epigenetic mechanisms involving the recruitment of lysine demethylases KDM6B and KDM4B, respectively.

A prompt diagnosis is particularly important for rare bone diseases such as primary thyroid osteosarcoma. In this Research Topic, Wang et al. identified *KMT2C* mutation as a potential diagnostic molecular marker. *KMT2C* encodes histone methyltransferase, which regulates gene transcription by modifying chromatin structure. KMT2C exerts a relevant role in embryonic development and cell proliferation. Interestingly, mutations of *KMT2C* were reported in 87% of osteosarcoma patients, suggesting its key role in bone carcinogenesis. Wang and coworkers described four variants of *KMT2C* mutations in a 72-year-old woman with primary thyroid osteosarcoma and discussed the relevance to consider osteosarcoma in the differential diagnosis of poorly differentiated and anaplastic thyroid malignancies with osteoid differentiation.

Csukasi et al. showed that mutations in the PTH receptor-1 (PTH1R) affect the differentiation of skeletal progenitors and lead to accumulation of fat or cartilage in bones in the autosomal dominant Jansen Metaphyseal Chondrodysplasia (JMC) and the autosomal recessive Blomstrand Chondrodysplasia (BOCD). JMC is a rare skeletal dysplasia characterized by abnormal endochondral bone formation and typically severe hypercalcemia due to delayed chondrocyte and bone formation (Gram et al., 1959; Silve and Jüppner, 2015); BOCD is a neonatal osteosclerotic dysplasia characterized by advanced endochondral bone maturation, very short limbs, dwarfism and prenatal lethality (Blomstrand et al., 1985; Jobert et al., 1998; Jüppner and Thakker, 2008; Hochberg, 2019). Csukasi et al. dissected the mechanisms by which skeletal progenitors differentiate towards bone or fat involving a pathway regulated by DEP-domain containing mTOR-interacting protein (DEPTOR) in a mTOR independent manner and TAZ protein.

The alteration of differentiation pathway leading to bone diseases was also studied by Xia et al. The authors described how *COG4* mutation affects chondrocyte differentiation in Saul-Wilson syndrome, a rare disease characterized by a distinct facial phenotype, short stature, brachydactyly, clubfoot deformities, cataracts and microcephaly. COG is a subunit of the conserved oligomeric Golgi (COG) complex which belongs to complexes associated with tethering containing helical rods (CATCHR) family. Xia et al. generated a cellular model of Saul-Wilson syndrome using the CRISPR knock-in technique in the chondrosarcoma cell line SW1353. They reported how *COG* mutation alters the trafficking and secretion of various proteoglycans, glycoproteins, collagens, ADAM metallopeptidases, MMPs, cathepsins and secreted factors including BMPs (Bone Morphogenetic Proteins), WNT proteins, growth factors and chemokines. These findings could explain the bone defects observed in patients. However, the mechanism behind the selectively affected protein trafficking by *COG4* variant and its implication in regulation of other signaling pathways is still unknown.

The papers published in this Research Topic pointed the attention on how the study of the molecular mechanisms regulating bone cells differentiation and activity is relevant to identify new molecular pathways involved in bone diseases pathogenesis. The identification of molecular markers could highlight novel therapeutic targets in order to restore the bone remodeling affected in skeletal disorders.
